# Pancreatic Cancer Screening in New-onset and Deteriorating Diabetes: Preliminary Results From the PANDOME Study

**DOI:** 10.1210/clinem/dgaf319

**Published:** 2025-05-29

**Authors:** Richard C Frank, Brian Shim, Tammy Lo, Deep Pandya, Thorsten L Krebs, Charles Ma, Daniel Labow, Jill Denowitz, Naveen Anand, Pramila Krumholtz, Kiyoe Sullivan, Mark Sanchez, Xiang Eric Dong, Ramanathan Seshadri, Antolin Trinidad, Dugho Jin

**Affiliations:** Department of Medicine, Division of Hematology/Oncology, Nuvance Health, Norwalk, CT 06856, USA; Department of Radiology, Nuvance Health, Danbury, CT 06810, USA; Department of Medicine, Division of Hematology/Oncology, Nuvance Health, Norwalk, CT 06856, USA; Department of Research & Innovation, Nuvance Health, Danbury, CT 06810, USA; Department of Radiology, Nuvance Health, Danbury, CT 06810, USA; Department of Medicine, Division of Hematology/Oncology, Nuvance Health, Norwalk, CT 06856, USA; Department of Surgery, Nuvance Health, Danbury, CT 06810, USA; Department of Medicine, Nuvance Health, Norwalk, CT 06856, USA; Department of Gastroenterology, Nuvance Health, Norwalk, CT 06856, USA; Department of Medicine, Division of Hematology/Oncology, Nuvance Health, Norwalk, CT 06856, USA; Department of Pathology, Nuvance Health, Norwalk, CT 06856, USA; Department of Research & Innovation, Nuvance Health, Danbury, CT 06810, USA; Department of Surgery, Nuvance Health, Danbury, CT 06810, USA; Department of Surgery, Nuvance Health, Danbury, CT 06810, USA; Department of Psychiatry, Nuvance Health, Danbury, CT 06810, USA; Department of Radiology, Nuvance Health, Danbury, CT 06810, USA

**Keywords:** new-onset diabetes, deteriorating diabetes, pancreatic cancer

## Abstract

**Objective:**

Pancreatic cancer (PC) has a high mortality rate due to the lack of early-stage detection strategies and lethality of advanced stage presentations. New-onset diabetes (NOD) in individuals ≥50 years old increases the risk 6- to 8-fold, making this group a target of early detection studies. There is also evidence that deteriorating diabetes (DD) may be a risk factor.

**Research design and methods:**

The study prospectively enrolled individuals ≥50 years with NOD or DD. Participants underwent magnetic resonance imaging/cholangiopancreatography, blood biobanking and anxiety/depression monitoring. Magnetic resonance imaging scans were scored as normal, benign-abnormal, suspicious, or incidental finding. Glycemic indices and physician referral patterns were captured.

**Results:**

Over a 6-year period, 625 individuals were screened and 109 enrolled, 97 (89%) had NOD, and 12 (11%) had DD. Compared to the NOD cohort, the DD cohort was older, had higher hemoglobin A1c levels (*P* = .02), greater weight loss (*P* = .0038), and insulin requirements (*P* < .0001). Four pancreas biopsies were performed for suspicious findings (3.6%), with a stage 1 pancreatic ductal adenocarcinoma identified in the DD group, corresponding to an overall detection rate of 0.9% (1/109). The detection rates of benign pancreatic abnormalities and incidental findings revealed no safety signals. Endocrinologists were the main referral source for the DD cohort (*P* < .001).

**Conclusion:**

Results from the PANDOME study thus far include the first reported screen-detected early-stage PC in a sporadic cohort. Our findings support the inclusion of a DD cohort in prospective PC screening studies in high-risk diabetes. Endocrinologists play an especially important role in the referral of individuals with DD.

By the year 2030, pancreatic ductal adenocarcinoma (PC) will surpass colorectal cancer as the second leading cause of cancer deaths in the United States with only 13 of every 100 affected individuals surviving 5 years (1). The lethality of PC is due in large measure to the lack of approved early detection methods: although the 5-year survival rate for stage 1 disease is 44%, fewer than 20% of affected individuals are diagnosed at this early stage (2). More commonly, PC presents at an advanced stage, in which average survivals are 10 months in fit individuals able to receive multiagent chemotherapy, but far shorter in those with advanced age and/or comorbidities that preclude such treatments (3, 4). Therefore, there is an urgent need to identify individuals at high risk of PC and develop effective early-stage detection strategies.

The pioneering work of the Cancer of the Pancreas Screening Study established that detection of early-stage PC in hereditary high-risk individuals (HRI) is both feasible and effective (5). This study used annual magnetic resonance imaging (MRI) or endoscopic ultrasound (EUS) in those with a strong family history of PC, half of whom carried a pathogenic variant in a PC susceptibility gene, such as *BRCA2*. The 5-year survival of screen-detected cancers was 73%. The Pancreatic Cancer Early Detection consortium is a prospective, international study that expands these efforts by obtaining serial biospecimens and regular imaging screening in HRI to promote the identification of serum and imaging markers of early PC (6). Similar PC screening trials have also been carried out in community settings (7, 8).

Despite the strides made in the early detection of hereditary PC, these advances have not extended to the >90% of cases which are considered sporadic. Risk factors for sporadic PC include older age, Black race, pancreatitis, smoking, alcohol, obesity, and diabetes (9). Among these, the highest risk exists in individuals ≥50 years of age who develop new-onset diabetes (NOD), which is associated with a 5.4-fold increased risk of PC within 1 year of NOD. In contrast, longstanding diabetes raises the risk 1.5-fold (10). Whereas the relationship between NOD and PC is well established (11-13), that between PC and deteriorating diabetes (DD) in those with longstanding, stable diabetes, has only recently been studied (14). Since loss of glycemic control may be an indicator of subclinical PC, successful screening strategies must consider this relationship across the entire spectrum of diabetes care.

Both diabetes and PC disproportionately impact Black Americans (15, 16). The capability to improve the rates of PC early detection among Black individuals with diabetes therefore holds the potential to meaningful reduce health disparities related to a PC diagnosis.

We opened the investigator-initiated, community-based PAncreatic cancer screening in New-onset and DeteriOrating diabetes MEllitus (PANDOME) study 3 years ago (17), after a 3-year NOD-only cohort. Here, we report on preliminary efficacy and safety results after the first 100 participants were enrolled. To our knowledge, this is the first report of a prospective, MRI-based imaging study in NOD (18) and the first to include a DD cohort.

## Research Design and Methods

The PANDOME Study is approved by the BRANY institutional review board and registered on ClinialTrials.gov (identifier NCT03937453). All study-related tests and evaluations were performed for free and supported by study funds. The study was conducted at Danbury and Norwalk Hospitals, 2 mid-sized community hospitals in Connecticut, part of Nuvance Health. Full inclusion/exclusion criteria can be found in the study protocol. Briefly, eligible participants were at least 50 years old with no contraindication to contrast-enhanced MRI/cholangiopancreatography (MRCP) and able to provide written informed consent. For the NOD cohort, because patients were not part of 1 closed health care system and prior results were not consistently available, we created 4 categories of eligibility: (1) NOD of confirmed duration: hemoglobin A1c (HbA1c) ≥ 6.5% within the past 12 months AND documentation of prior normal fasting plasma glucose, oral glucose tolerance test, or HbA1c levels within the past 24 months; (2) NOD of unconfirmed duration: HbA1c ≥ 7.0% within the past 12 months BUT no prior record of normal fasting plasma glucose, HbA1c, or oral glucose tolerance test within the past 24 months; (3) Transition from prediabetes to NOD: a rise from prediabetes HbA1c levels (5.7-6.4%) to ≥6.5% provided the increment was at least 0.5%; and (4) NOD with 1 first-degree relative with PC: HbA1c ≥ 6.5% within the past 12 months, that is of confirmed or unconfirmed duration, in an individual with 1 first-degree relative with PC. DD cohort eligibility included a history of longstanding diabetes (>2 years) and a > 2% increase in HbA1c within the past 6 months, not associated with weight gain or diabetes medication noncompliance.

### Recruitment

Recruitment of NOD cohort participants involved physician engagement (through in person meetings, emails, and direct mailing of flyers), public outreach through various social media platforms, and institutional review board approved data mining of the electronic medical record. Primary care physicians were notified of potential participants identified by data mining and these individuals were contacted via letters and telephone calls unless their primary care physician advised against their participation. All potential DD cohort participants were referred by physicians.

### Study Design

Every 6 months for 3 years, participants underwent a history and physical examination by the study advanced practice registered nurse, provided blood specimens for biobanking and had anxiety and depression assessed by the Hospital Anxiety and Depression Scale (19). Participants were contacted by phone annually through year 5 to monitor for the development of PC.

### Image Interpretation and Reporting

Gadolinium-enhanced MRI/MRCP was performed using both 1.5-T and 3-T MRI scanners, with a small field of view protocol dedicated to pancreatic imaging. All MRI scans were initially read by a body radiologist and then reviewed again at a multidisciplinary committee consisting of fellowship-trained body radiologists as well as interventional gastroenterologists and medical and surgical oncologists specializing in PC.

### ENDPAC Score Calculation

The Enriching New-onset Diabetes for PAncreatic Cancer (ENDPAC) scores were calculated using the original model for NOD and modified model for DD, as developed by Sharma et al (20) and Chen et al (21), respectively. The following parameters were included: (1) change in blood glucose from the year before the diagnosis of NOD; (2) change in weight from the year before NOD/DD; and (3) age at the time of NOD/DD. Single plasma glucose values at the time of NOD (predominantly fasting blood glucose) and HbA1c values at the time of DD were compared to those between 3 and 18 months before to calculate the glucose score. If blood glucose was not available, then estimated average glucose was calculated using the formula 28.7 × HbA1c − 46.7 (20). ENDPAC scores could not be calculated for 38 participants, who were enrolled under DM of unconfirmed duration criteria because prior glucose data were missing.

### Statistical Analysis

Descriptive statistics were used to summarize baseline demographic characteristics, clinical variables, and outcomes. Continuous variables were by Wilcoxon rank-sum test and categorical variables are presented as numbers and percentage and analyzed by chi-square. The disparities in ENDPAC scores among the NOD and DD groups were assessed using the Wilcoxon rank-sum test, whereas the distribution of ENDPAC score thresholds was analyzed using the chi-square test, applying a cutoff of ≥3 for the NOD cohort and ≥5 for the DD cohort to define high-risk classifications (20, 21). A *P* < .05 was deemed statistically significant.

### Data and Resource Availability

The datasets generated or analyzed during this study are available from the corresponding author upon reasonable request.

## Results

### Participants and Referral Patterns

Over a 6-year period, 625 individuals were screened, 410 deemed eligible and 109 consented and ultimately underwent MRI (Supplementary Fig. S1) (41). The average time on study was 31.6 months. Among enrollees, 84% were referred by their physicians, 11% through data mining, and 5% were self-referred. Reasons for declining to participate included: (1) lack of interest; (2) too great a distance to the study sites; (3) anxiety related to gadolinium contrast; (4) anxiety related to potential incidental findings; (5) lack of available time; and (6) a feeling that research related to pancreatic cancer was depressing.

The demographic and clinical characteristics of the participants was similar between the NOD (n = 97) and DD (n = 12) cohorts with the exception that DD was older than NOD (median age, 68 vs 61 years old; *P* = .03) (Table 1). Nearly 83% of participants were White and 17.4% being either Black, Asian, Latino, or Native American. Regarding patterns of referral, 95% of primary care physician referrals were for the NOD cohort, whereas endocrinologists referred equally to NOD and DD. Overall, 67% of the DD cohort was referred by endocrinologists (*P* < .0001) (Table 1).

**Table 1.** Baseline demographics and physician referral patterns of patients with new-onset diabetes (NOD) and deteriorating diabetes (DD)

Category	Subcategory	NOD (n = 97)	DD (n = 12)	*P*
Age	Median (range)	61.0 (50-78)	68.0 (51-83)	.0341*
Sex	Female	62 (63.9%)	7 (58.3%)	.705
	Male	35 (36.1%)	5 (41.7%)	
Ethnicity	Black	8 (8.2%)	1 (8.3%)	.9049
	Asian	4 (4.1%)	1 (8.3%)	
	Hispanic	4 (4.1%)	0 (0.0%)	
	Native American	1 (1.0%)	0 (0.0%)	
	White	80 (82.5%)	10 (83.3%)	
Tobacco use	Never	46 (47.4%)	3 (25.0%)	.055
	Past	45 (46.4%)	6 (50.0%)	
	Present	6 (6.2%)	3 (25.0%)	
Alcohol use	None	65 (67.0%)	5 (41.7%)	.3894
	Light	9 (9.3%)	2 (16.7%)	
	Moderate	4 (4.1%)	1 (8.3%)	
	Heavy	19 (19.6%)	4 (33.3%)	
Exercise	Light	54 (55.7%)	4 (33.3%)	.1315
	Vigorous	5 (5.2%)	0 (0.0%)	
	Moderate	23 (23.7%)	3 (25.0%)	
	Sedentary	15 (15.5%)	5 (41.7%)	
FDR with DM	Yes	57 (58.8%)	9 (75.0%)	.2776
	No	40 (41.2%)	3 (25.0%)	
FDR with PC	No	15 (15.5%)	1 (8.3%)	.5103
	Yes	82 (84.5%)	11 (91.7%)	
BMI	Overweight	13 (13.4%)	2 (16.7%)	.9485
	Obese	35 (36.1%)	4 (33.3%)	
	Normal	49 (50.5%)	6 (50.0%)	
ECOG score	0	78 (80.4%)	10 (83.3%)	.8088
	1	19 (19.6%)	2 (16.7%)	
Referral	Endocrinologists	7 (7.2%)	8 (66.7%)	Endocrinology vs others *P* value −.0010* Endocrinology vs primary care *P* value −<.0001*
	Others*^a^*	24 (24.7%)	2 (16.7%)	
	Primary care	66 (68.0%)	2 (16.7%)	

Abbreviations: BMI, body mass index; ECOG, Eastern Cooperative Oncology Group; FDR, first-degree relative; PC, pancreatic cancer.

*Statistically significant.

^*a*^Other sources of referral: dieticians, patient/family, medical oncologist, electronic medical record.

Among the 4 categories of NOD cohort eligibility, 33% had NOD of unconfirmed duration, 32% transition from prediabetes, 16% NOD of confirmed duration, and 10% NOD plus 1 first-degree relative with PC. The average duration of diabetes for those in DD was 9 years (range, 2-20; only 1 individual <3 years duration).

### Glycemic Indices of NOD/DD Cohorts

There were significant differences in glycemic parameters between the NOD and DD cohorts as indicated in Table 2. As expected, HbA1c values were 1.6% higher for the DD cohort (*P* = .02) as a > 2% spike was required for study entry. Insulin was initiated upon diagnosis of NOD or DD in 10% and 67%, respectively (*P* < .0001). DD participants lost an average of 6.3 kg compared to 0.6 kg for NOD participants (*P* = .0038).

**Table 2.** Glycemic characteristics of patients with new-onset diabetes (NOD) and deteriorating diabetes (DD)

Index		NOD (n = 97)	DD (n = 12)	*P*
HbA1c at entry (%)	Mean ± SD	9.4 ± 2.5	11.0 ± 2.0	.0204*
BG at entry (mg/dL)	Mean ± SD	256.8 ± 125.9	331.1 ± 150.0	.0680
BG 1 y prior (mg/dL)	Mean ± SD	119.6 ± 27.0	141.0 ± 36.0	.0287*
Insulin initiated, n (%)	Yes	10 (10.3%)	8 (66.7%)	<.0001*
	No	87 (89.7%)	4 (33.3%)	
Weight at entry (kg)	Mean ± SD	92.1 ± 19.4	82.3 ± 15.9	.1054
Weight 1 y prior (kg)	Mean ± SD	93.6 ± 20.8	88.6 ± 14.8	.4993
Δ Weight	Mean ± SD	−0.60 ± 5.5	−6.3 ± 6.2	.0038*
ENDPAC score	Mean ± SD	1.5 ± 4.3	7.92 ± 3.2	<.0001*
High risk*^a^*	Yes	21 (35.6%)	9 (75.0%)	.0118*
	No	38 (64.4%)	3 (25%)	

Abbreviations: BG, blood glucose; ENDPAC, Enriching New-Onset Diabetes for Pancreatic Cancer; HbA1c, hemoglobin A1c.

*Statistically significant.

^*a*^High-risk ENDPAC scores: ≥3 for NOD and ≥5 for DD (20, 21).

Given the much greater prevalence of NOD compared to PC in the general population, a risk stratification model called ENDPAC was developed to enrich for NOD at high risk of PC, that considers 3 factors: age, change in weight and change in blood glucose at the time of NOD (20). This model was subsequently modified to accommodate the use of HbA1c (instead of fasting glucose) values, which is more applicable to individuals with longstanding diabetes for whom HbA1c levels are routinely measured (21). A high-risk ENDPAC score has been associated with an estimated 3.6% probability of pancreatic cancer within 3 years (20). Using these models, the DD cohort had a significantly higher percentage of high-risk individuals than the NOD cohort (75% vs 35.6%, *P* = .0118) [Table 2; Supplementary Fig. S2A/B (41)].

### Anxiety and Depression Testing

Longitudinal trends in anxiety and depression scores using the Hospital Anxiety and Depression Scale standardized test (19) did not reveal statistically significant changes during the period analyzed although both saw declines from baseline over the first 18 to 24 months of study participation (Supplementary Fig. S3) (41). Participation in the pancreatic cancer screening study was not associated with an increase in anxiety or depression. This is consistent with other studies indicating that screening per se does not necessarily increase levels of anxiety and depression in this population, and that repeated screening is of low psychological burden (22).

### MRI/MRCP Results

As shown in Table 3, among 109 participants who underwent pancreatic MRI, >50% had small cystic lesions, which were predominantly unilocular, with an average cyst size of 6 mm (range, 2-37 mm). EUS was performed on 7 participants (6.4%) with 4 (3.7%) requiring biopsy. Extrapancreatic incidental findings were detected in 8.2% (9/109), with 2 cases requiring biopsy (1.8%), revealing 1 new diagnosis of follicular lymphoma and 1 diagnosis of recurrent lymphoma (Supplementary Table S1) (41). Of the 4 pancreas biopsies performed, 1 case of adenocarcinoma was diagnosed (see the following).

**Table 3.** Summary of pancreatic MRI screening findings

	NOD (n = 97)	DD (n = 12)
Normal pancreas	41 (42.3%)	4 (33.3%)
Pancreas with cyst(s)	55 (56.7%)	8 (66.7%)
Number of cysts		
1-2	36 (37.1%)	4 (33.3%)
≥3	19 (19.6%)	4 (33.3%)
Average cyst size	6.1 mm	6.2 mm
Unilocular	50 (90.9%)	6 (75.0%)
Multilocular	5 (9.1%)	2 (25.0%)
Ductal dilation		
Yes	4 (4.1%)	1 (8.3%)
No	93 (95.9%)	11 (91.7%)
Endoscopic ultrasound performed	6 (6.2%)	2 (16.7%)
Incidental findings requiring biopsy*^a^*	2 (2.1%)	0
Pancreatic cancers	0	1 (8.3%)

Abbreviations: DD, deteriorating diabetes; MRI, magnetic resonance imaging; NOD, new-onset diabetes.

^*a*^Two incidental findings required biopsy, both revealed non-Hodgkin's lymphoma.

### Case Report

**Figure 1.**
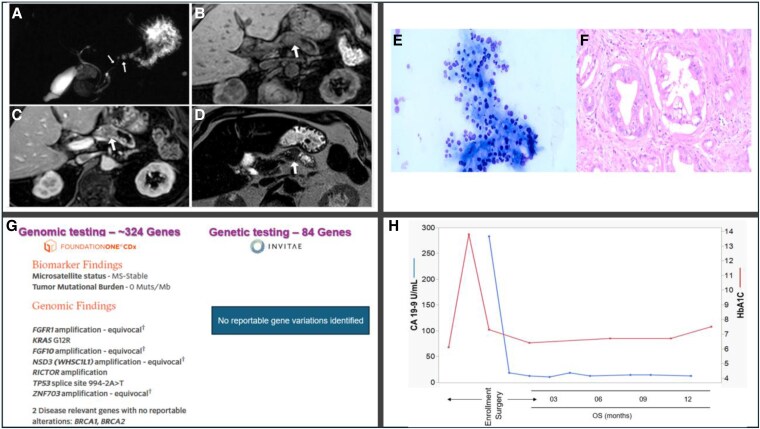
Clinical and pathologic features of a screen-detected stage 1B pancreatic ductal adenocarcinoma in an 82-year-old woman with deteriorating diabetes. (A) MRCP image demonstrates dilation of pancreatic duct (arrows). (B) Precontrast T1-weighted MRI scan, and (C) contrast-enhanced T1 image demonstrate hypo-enhancing mass at the body (arrows) and dilation of the pancreatic duct within the tail region. (D) T2-weighted image shows dilated pancreatic tail which abruptly transitions at the level of the pancreatic body (arrow). (E) Diagnostic aspiration cytology obtained by endoscopic ultrasound shows clusters of malignant cells. (F) Sections from distal pancreatectomy showing infiltrating ductal adenocarcinoma. (G) Tumor genomic profiling showing canonical mutations in KRAS, TP53, and others. (H) Tumor marker CA 19-9 and HbA1c values pre- and postsurgery.

An active, independent 82-year-old woman with a 10-year history of stable diabetes on metformin presented to the hospital with malaise, polyuria/polydipsia, a 20-pound weight loss, glucose >500 mg/dL, and HbA1c 13.8%, increased from 6.1% 3 months earlier. Her internist referred her to an endocrinologist who initiated insulin therapy and referred her for the PANDOME study within 3 months of DD. Her ENDPAC score was 11. A metastatic work-up was negative. The CA 19-9 was 283 U/mL (normal, <35 U/mL). MRI scans, pancreas tumor pathology, tumor genomic profiling, and CA 19-9 values before and after surgery are shown in Fig. 1A-1H. Germline genetic testing was normal. She underwent distal pancreatectomy/splenectomy and was discharged from the hospital 3 days later. Pathology revealed ductal adenocarcinoma, 3 cm with clear margins, 0/15 lymph nodes involved, pT2N0M0, Stage 1B. She received 6 months of dose-reduced adjuvant 5-fluorouracil, leucovorin, irinotecan, and oxaliplatin chemotherapy and remains cancer-free at 18 months. Her diabetes medications include metformin and insulin and her HbA1c values have remained ≤7.5% (Fig. 1H).

## Discussion

Pancreatic cancer is a global public health crisis, accounting for 511 000 new cases and 467 000 deaths in 2022 (23). Since survival is directly related to stage at diagnosis, novel approaches are urgently needed to detect early-stage PC in nonhereditary (sporadic) high-risk populations. In this prospective, MRI-based imaging study in individuals with NOD or DD, we present preliminary evidence demonstrating the safety of this approach and the successful detection and treatment of a stage 1 PC in an individual with DD. To our knowledge, these results represent several firsts: (1) the first report of a screen-detected, early-stage PC in a sporadic cohort; (2) the first to use MRI-based screening in individuals with NOD; and (3) the first prospective study to include a DD cohort.

The first case report of diabetes preceding PC was in 1833 by R. Bright from Guy's Hospital, London (24). In 1969, Karmody and Kyle analyzed the prevalence and chronology of diabetes in 256 PC cases (>60 years old), at the Royal Infirmary, Scotland. They noted that 80% of PC-related diabetes occurred within one year of PC and that “It is in people of this age that glycosuria and hyperglycemia should be regarded as possible early signs of carcinoma of the pancreas, especially if the blood glucose level is difficult to control and there is marked loss of weight” (25). In 1994, Noy and Bilezikian reanalyzed prior studies, noting prevalence rates of diabetes in PC as high as 40%, concluding that the greatest risk of PC was in those who had “sudden” or “new-onset diabetes” (within 1 year of PC). They concluded that NOD which precedes PC is caused by an occult cancer and might serve as a harbinger of subclinical PC (26).

In 2005, Chari et al reported that 0.85% of individuals with NOD older than 50 years in Olmstead County, Minnesota, developed PC within 3 years (13). This represented a 6- to 8-fold increased risk of PC in this group compared to the general population. In 2018, the consortium for the study of chronic pancreatitis, diabetes, and pancreatic cancer initiated a prospective study to establish a NOD cohort to validate these findings, among other goals (27). A pilot study using multidetector computerized tomography was performed on 93 NOD participants (18). Preliminary findings included a high rate of extrapancreatic findings (56%) and 1 PC (1.1%) that was unfortunately stage 4 with liver metastases. Refinement of this early detection initiative includes incorporation of the ENDPAC risk-stratification algorithm (28).

We initiated a NOD-only study more than 6 years ago and selected MRI/MRCP as the screening modality due to its proven success in large PC screening programs of hereditary HRI (5). Among our 97 NOD participants, we did not detect a case of PC, which is likely attributable to the low ENDPAC scores of this cohort. Regarding safety and cost, MRI/MRCP detected small, simple pancreatic cysts in >50% of participants, which is not unexpected but will need to be followed per standard guidelines (29). Nine participants (8.2%) required additional procedures, such as EUS or biopsy of incidental findings, which were uncomplicated. Although procedures increase costs, they were of high yield as 1 of 4 pancreas biopsies revealed cancer and both biopsies for incidental findings yielded other cancer diagnoses. As study accrual grows, the true cost-effectiveness of this approach will be clarified.

Based on clinical observations and past publications linking loss of diabetes control in longstanding diabetes to the development of PC (30, 31), we added a DD cohort to a revised study, termed PANDOME, 4 years ago. After discussions with endocrinologists, we set the criterion for DD as a > 2% increase in HbA1c within 6 months of enrollment, in the absence of weight gain or medication noncompliance. Recent analyses of large health system databases support these decisions. Defining DD as the intensification of an antihyperglycemic regimen, Mueller et al analyzed the United Kingdom Clinical Practice Research Dataline GOLD and showed that a HbA1c increase of ≥2% in longstanding diabetes was commonly found in those who developed PC. They also found that body weight decreased (1.9% ± 6.4%) in the PC group, in contrast to weight gain in the non-PC group (32). In 2024, Ali et al analyzed a population-based cohort of Australian woman with unstable and stable diabetes and reported that unstable diabetes was associated with a 2.5-fold increased risk of PC overall, rising to 5.76-fold within the first 3 months (14). Thus, the hazard ratios for PC related to both NOD and DD are greater than 5-fold.

Although our DD cohort is small, it did capture a stage 1B PC. The detection of early stage, lymph node-negative PC is a central goal of screening programs (33). This individual was elderly, lost weight, and had a 7% spike in her HbA1c, which required the addition of insulin to her diabetes regimen; her ENDPAC score was 11, which is the maximum (high-risk ≥5). In studies of DD-related PC, the requirement for insulin was associated with a hazard ratio of 7.67 at 3 months (14). Since age, glycemic change, and weight loss are the 3 factors considered in the ENDPAC algorithm (20, 21), our definition of DD enriches for individuals with high ENDPAC scores, who are at the greatest risk for PC. We suggest that the ENDPAC model be further studied and validated for use in individuals with DD. Our results suggest that older individuals, with abrupt glycemic worsening and elevated ENDPAC scores, may represent an enriched subgroup for early PC detection. Although our study was not powered to determine subgroup-specific screening efficacy the identification of a stage 1B cancer suggests that focusing on individuals with high-risk ENDPAC scores, regardless of diabetes classification, may enhance early-stage PC detection.

The mechanism of PC-induced diabetes (pancreatogenic type 3c) has not been fully elucidated but is thought to be a paraneoplastic phenomenon, related to PC cell secretion of extracellular vesicles that mediate insulin resistance and B-cell dysfunction (34-36). This hypothesis is supported by observations that NOD resolves in many patients after primary tumor resection (37) and glycemic control improves with successful treatment of unresectable disease (38). The use of circulating extracellular vesicles to develop a blood-based, early diagnostic biomarker of PC is an area of intensive investigation (39, 40). The application of such a biomarker to individuals with high-risk NOD and DD could provide an important enrichment filter of those who should be screened.

The strengths of our study include: (1) the ability to author, fund, and execute an investigator-initiated PC screening study in a real-world, community setting, close to where most participants reside; (2) the decision to add a DD cohort, which led to detection of a stage 1 PC; (3) our chosen HbA1c eligibility requirement for the DD cohort (>2%), which subsequently aligned with published analyses; and (4) involvement of endocrinologists as well as primary care providers as sources of referrals.

The main weaknesses of the study include: (1) low accrual rates, attributed to realignments in our health care network and electronic medical record and the high rate of declination by potential participants. Efforts to rectify these issues as well as partner with another health care network are in progress; (2) selection bias, potentially leading to lower detections rates. Since the majority of study participants were referred by primary care physicians or endocrinologists, it is possible that patients who raised higher levels of clinical suspicion for PC were managed outside of the study protocol, leading to referral bias and an underestimation of cancer detection rates. This highlights a key challenge in community-based screening efforts and underscores the need for standardized referral pathways in future studies; and (3) low ethnicity heterogeneity, despite the known increased risk of PC in Black individuals. Since our cohort was predominantly White (83%), further recruitment efforts will focus on improving representation of underrepresented communities by working with community health clinics, social organizations, and local foundations.

In conclusion, preliminary results from the PANDOME study support further MRI-based PC screening research efforts in individuals with NOD and DD. The results presented are insufficient to support the routine imaging of individuals with NOD or DD in routine practice. Refinement and harmonization of high-risk criteria, coupled with robust enrollment into clinical trials, hold the promise of delivering new diabetes-specific PC screening guidelines that may finally move the needle towards cure in this lethal disease.

## Data Availability

The data sets generated during the current study are not publicly available but are available from the corresponding author on reasonable request.

## Clinical trial registration

ClinicalTrials.gov ID: NCT03937453

## Abbreviations

DD: deteriorating diabetes
ENDPAC: Enriching New-onset Diabetes for PAncreatic Cancer
EUS: endoscopic ultrasound
HbA1c: hemoglobin A1c
HRI: high-risk individual
MRCP: magnetic resonance imaging/cholangiopancreatography
MRI: magnetic resonance imaging
NOD: new-onset diabetes
PANDOME: PAncreatic cancer screening in New-onset and DeteriOrating diabetes MEllitus
PC: pancreatic ductal adenocarcinoma

## References

[1] Rahib L, Smith BD, Aizenberg R, Rosenzweig AB, Fleshman JM, Matrisian LM. Projecting cancer incidence and deaths to 2030: the unexpected burden of thyroid, liver, and pancreas cancers in the United States. Cancer Res. 2014;74(11):2913‐2921. 24840647 10.1158/0008-5472.CAN-14-0155

[2] Siegel RL, Giaquinto AN, Jemal A. Cancer statistics, 2024. CA Cancer J Clin. 2024;74(1):12‐49. 38230766 10.3322/caac.21820

[3] Wainberg ZA, Melisi D, Macarulla T, et al NALIRIFOX versus nab-paclitaxel and gemcitabine in treatment-naive patients with metastatic pancreatic ductal adenocarcinoma (NAPOLI 3): a randomised, open-label, phase 3 trial. Lancet. 2023;402(10409):1272‐1281. 37708904 10.1016/S0140-6736(23)01366-1 PMC11664154

[4] Tabernero J, Chiorean EG, Infante JR, et al Prognostic factors of survival in a randomized phase III trial (MPACT) of weekly nab-paclitaxel plus gemcitabine versus gemcitabine alone in patients with metastatic pancreatic cancer. Oncologist. 2015;20(2):143‐150. 25582141 10.1634/theoncologist.2014-0394 PMC4319641

[5] Dbouk M, Katona BW, Brand RE, et al The multicenter cancer of pancreas screening study: impact on stage and survival. J Clin Oncol. 2022;40(28):3257‐3266. 35704792 10.1200/JCO.22.00298 PMC9553376

[6] Zogopoulos G, Haimi I, Sanoba SA, et al The pancreatic cancer early detection (PRECEDE) study is a global effort to drive early detection: baseline imaging findings in high-rsk individuals. J Natl Compr Canc Netw. 2024;22(3):158‐166. 38626807 10.6004/jnccn.2023.7097 PMC12344727

[7] Kandiah J, Lo T, Jin D, et al A community-based pancreatic cancer screening study in high-risk individuals: preliminary efficacy and safety results. Clin Transl Gastroenterol. 2022;13(8):e00516. 35854467 10.14309/ctg.0000000000000516 PMC9400932

[8] Raff JP, Cook B, Jafri FN, et al Successful pancreatic cancer screening among individuals at elevated risk using endoscopic ultrasound and magnetic resonance imaging: a community hospital experience. Pancreas. 2022;51(10):1345‐1351. 37099777 10.1097/MPA.0000000000002182

[9] Hu JX, Zhao CF, Chen WB, et al Pancreatic cancer: a review of epidemiology, trend, and risk factors. World J Gastroenterol. 2021;27(27):4298‐4321. 34366606 10.3748/wjg.v27.i27.4298 PMC8316912

[10] Pereira SP, Oldfield L, Ney A, et al Early detection of pancreatic cancer. Lancet Gastroenterol Hepatol. 2020;5(7):698‐710. 32135127 10.1016/S2468-1253(19)30416-9 PMC7380506

[11] Aggarwal G, Rabe KG, Petersen GM, Chari ST. New-onset diabetes in pancreatic cancer: a study in the primary care setting. Pancreatology. 2012;12(2):156‐161. 22487526 10.1016/j.pan.2012.02.003 PMC4348043

[12] Chari ST, Leibson CL, Rabe KG, Ransom J, de Andrade M, Petersen GM. Probability of pancreatic cancer following diabetes: a population-based study. Gastroenterology. 2005;129(2):504‐511. 16083707 10.1053/j.gastro.2005.05.007 PMC2377196

[13] Chari ST, Leibson CL, Rabe KG, et al Pancreatic cancer-associated diabetes mellitus: prevalence and temporal association with diagnosis of cancer. Gastroenterology. 2008;134(1):95‐101. 18061176 10.1053/j.gastro.2007.10.040 PMC2271041

[14] Ali S, Coory M, Donovan P, et al Association between unstable diabetes mellitus and risk of pancreatic cancer. Pancreatology. 2024;24(1):66‐72. 38000983 10.1016/j.pan.2023.11.009

[15] Menke A, Casagrande S, Geiss L, Cowie CC. Prevalence of and trends in diabetes among adults in the United States, 1988-2012. JAMA. 2015;314(10):1021‐1029. 26348752 10.1001/jama.2015.10029

[16] Ashktorab H, Kupfer SS, Brim H, Carethers JM. Racial disparity in gastrointestinal cancer risk. Gastroenterology. 2017;153(4):910‐923. 28807841 10.1053/j.gastro.2017.08.018 PMC5623134

[17] Frank RC, Lo T, Jin D, et al A pancreatic cancer screening study in new-onset and deteriorating diabetes mellitus (PANDOME study). J Clin Oncol. 2021;39(15_suppl):TPS10607.

[18] Wu BU, Lustigova E, Chen Q, et al Imaging of the pancreas in new-onset diabetes: a prospective pilot study. Clin Transl Gastroenterol. 2022;13(6):e00478. 35333778 10.14309/ctg.0000000000000478 PMC9236602

[19] Moseholm E, Rydahl-Hansen S, Overgaard D, et al Health-related quality of life, anxiety and depression in the diagnostic phase of suspected cancer, and the influence of diagnosis. Health Qual Life Outcomes. 2016;14(1):80. 27206557 10.1186/s12955-016-0484-9 PMC4873991

[20] Sharma A, Kandlakunta H, Nagpal SJS, et al Model to determine risk of pancreatic cancer in patients with new-onset diabetes. Gastroenterology. 2018;155(3):730‐739.e3. 29775599 10.1053/j.gastro.2018.05.023 PMC6120785

[21] Chen W, Zhou B, Luong TQ, et al Prediction of pancreatic cancer in patients with new onset hyperglycemia: a modified ENDPAC model. Pancreatology. 2024;24(7):1115‐1122. 39353843 10.1016/j.pan.2024.09.015

[22] Konings IC, Sidharta GN, Harinck F, et al Repeated participation in pancreatic cancer surveillance by high-risk individuals imposes low psychological burden. Psychooncology. 2016;25(8):971‐978. 26632416 10.1002/pon.4047

[23] Bray F, Laversanne M, Sung H, et al Global cancer statistics 2022: GLOBOCAN estimates of incidence and mortality worldwide for 36 cancers in 185 countries. CA Cancer J Clin. 2024;74(3):229‐263. 38572751 10.3322/caac.21834

[24] Bright R . Cases and observations connected with disease of the pancreas and duodenum. Med Chir Trans. 1833;18(P1)(Pt 1):1‐56. 10.1177/09595287330180p102 PMC2116550 20895598

[25] Karmody AJ, Kyle J. The association between carcinoma of the pancreas and diabetes mellitus. Br J Surg. 1969;56(5):362‐364. 5781048 10.1002/bjs.1800560512

[26] Noy A, Bilezikian JP. Clinical review 63: diabetes and pancreatic cancer: clues to the early diagnosis of pancreatic malignancy. J Clin Endocrinol Metab. 1994;79(5):1223‐1231. 7962312 10.1210/jcem.79.5.7962312

[27] Maitra A, Sharma A, Brand RE, et al A prospective study to establish a new-onset diabetes cohort: from the consortium for the study of chronic pancreatitis, diabetes, and pancreatic cancer. Pancreas. 2018;47(10):1244‐1248. 30325864 10.1097/MPA.0000000000001169 PMC6432934

[28] Chari ST, Maitra A, Matrisian LM, et al Early detection initiative: a randomized controlled trial of algorithm-based screening in patients with new onset hyperglycemia and diabetes for early detection of pancreatic ductal adenocarcinoma. Contemp Clin Trials. 2022;113:106659. 34954100 10.1016/j.cct.2021.106659 PMC8844106

[29] Gonda TA, Cahen DL, Farrell JJ. Pancreatic cysts. N Engl J Med. 2024;391(9):832‐843. 39231345 10.1056/NEJMra2309041

[30] Sadr-Azodi O, Gudbjörnsdottir S, Ljung R. Pattern of increasing HbA1c levels in patients with diabetes mellitus before clinical detection of pancreatic cancer—a population-based nationwide case-control study. Acta Oncol. 2015;54(7):986‐992. 25734801 10.3109/0284186X.2015.1006402

[31] Mueller AM, Meier CR, Jick SS, Schneider C. The potential of glycemic control and body weight change as early markers for pancreatic cancer in patients with long-standing diabetes mellitus: a case–control study. Pancreas. 2018;47(7):807‐815. 29975346 10.1097/MPA.0000000000001085

[32] Mueller AM, Meier CR, Jick SS, Schneider C. Characterization of the deterioration of diabetes control in patients with a subsequent diagnosis of pancreatic cancer: a descriptive study. Pancreatology. 2022;22(3):387‐395. 35314354 10.1016/j.pan.2022.03.012

[33] Mazer BL, Lee JW, Roberts NJ, et al Screening for pancreatic cancer has the potential to save lives, but is it practical? Expert Rev Gastroenterol Hepatol. 2023;17(6):555‐574. 37212770 10.1080/17474124.2023.2217354 PMC10424088

[34] Javeed N, Sagar G, Dutta SK, et al Pancreatic cancer-derived exosomes cause paraneoplastic beta-cell dysfunction. Clin Cancer Res. 2015;21(7):1722‐1733. 25355928 10.1158/1078-0432.CCR-14-2022 PMC4383684

[35] Wang L, Zhang B, Zheng W, et al Exosomes derived from pancreatic cancer cells induce insulin resistance in C2C12 myotube cells through the PI3K/Akt/FoxO1 pathway. Sci Rep. 2017;7(1):5384. 28710412 10.1038/s41598-017-05541-4 PMC5511275

[36] Wlodarczyk B, Durko L, Walczak K, Talar-Wojnarowska R, Malecka-Wojciesko E. Select endocrine disorders and exosomes in early PDAC diagnosis. Int J Mol Sci. 2024;25(22):12159. 39596226 10.3390/ijms252212159 PMC11594802

[37] Pannala R, Leirness JB, Bamlet WR, Basu A, Petersen GM, Chari ST. Prevalence and clinical profile of pancreatic cancer-associated diabetes mellitus. Gastroenterology. 2008;134(4):981‐987. 18395079 10.1053/j.gastro.2008.01.039 PMC2323514

[38] Alpertunga I, Sadiq R, Pandya D, et al Glycemic control as an early prognostic marker in advanced pancreatic cancer. Front Oncol. 2021;11:571855. 33718132 10.3389/fonc.2021.571855 PMC7947820

[39] Nakamura K, Zhu Z, Roy S, et al An exosome-based transcriptomic signature for noninvasive, early detection of patients with pancreatic ductal adenocarcinoma: a multicenter cohort study. Gastroenterology. 2022;163(5):1252‐1266.e2. 35850192 10.1053/j.gastro.2022.06.090 PMC9613527

[40] Hinestrosa JP, Kurzrock R, Lewis JM, et al Early-stage multi-cancer detection using an extracellular vesicle protein-based blood test. Commun Med (Lond). 2022;2(1):29. 35603292 10.1038/s43856-022-00088-6 PMC9053211

[41] Frank RC, Shim B, Lo T, et al Supplementary Figures for “Pancreatic Cancer Screening in New-Onset and Deteriorating Diabetes: Preliminary Results from the PANDOME Study”. Figshare. Dataset. 2025. 10.6084/m9.figshare.29224817.v1 PMC12712999 40439123

